# Quadricuspid aortic valve not discovered by transthoracic echocardiography

**DOI:** 10.1186/1476-7120-4-41

**Published:** 2006-11-07

**Authors:** Magnus Dencker, Martin Stagmo

**Affiliations:** 1 Dept of Clinical Sciences, Unit of Clinical Physiology and Nuclear Medicine, University Hospital MAS, Lund University, Malmö, Sweden; 2 Dept of Cardiology, University Hospital MAS, Lund University, Malmö, Sweden

## Abstract

**Background:**

Quadricuspid aortic valve is a rare congenital heart defect. Several different anatomical variations of a quadricuspid aortic valve has been described and aortic regurgitation is the predominant valvular dysfunction associated with quadricuspid aortic valve.

**Case presentation:**

A 68-year-old woman presented with almost a years history of increasing dyspnoea on exertion. The patient have had two previous transthoracic echocardiographic exams in the last six years and they had only documented moderate aortic regurgitation. Transoesophageal echocardiography displayed a rare case of quadricuspid aortic valve with three cusps of equal size and one larger cusp. The malformation was associated with severe aortic regurgitation.

**Conclusion:**

Liberal use of transoesophageal echocardiography is often warranted if optimal display of valvular morphology is desired.

## Background

Quadricuspid aortic valve is a rare congenital heart defect. It has in the past been an incidental finding at open heart surgery or at autopsy. There has in recent years been a few case report, with the increasing use of echocardiography [1-8].

## Case presentation

**Figure 1:**
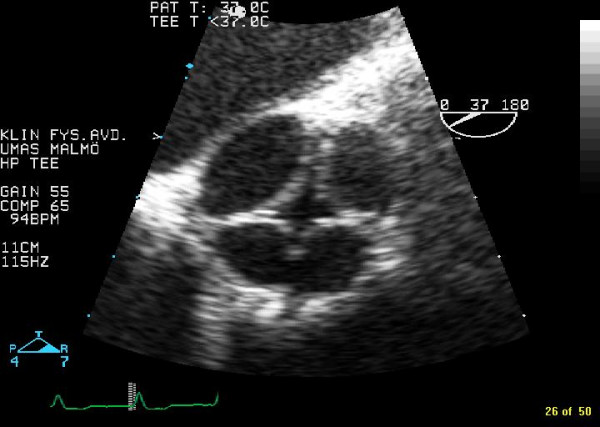
Transoesophageal images, in diastole, of a quadricuspid aortic valve with three cusps of equal size and one larger cusp.

**Figure 2:**
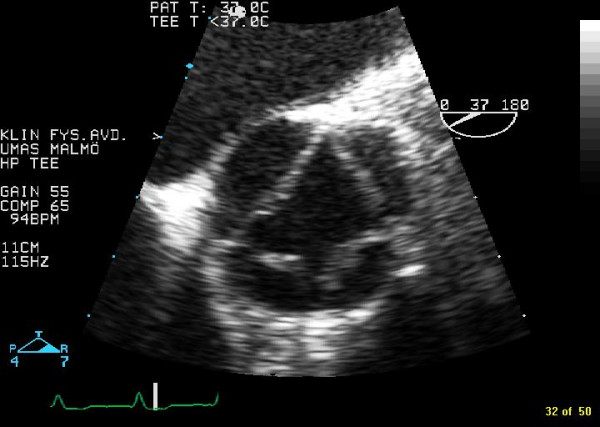
Transoesophageal images of a quadricuspid aortic valve in systole.

**Figure 3:**
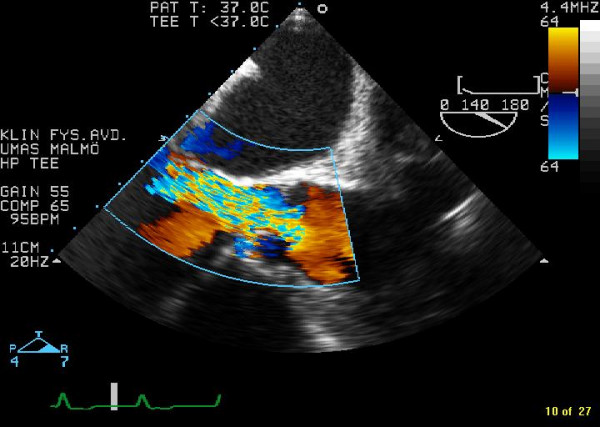
Transoesophageal Colour-Doppler image of severe aortic regurgitation associated with the quadricuspid aortic valve in this case.

A 68-year-old woman presented with almost a years history of increasing dyspnoea on exertion. On physical examination, her blood pressure was 200/90 mm Hg and a diastolic murmur of grade 5/6 was heard at the left sternal border. ECG displayed, sinusrhythm with high QRS-voltage and ST-T configuration consistent with left ventricular hypertrophy. Chest X ray was normal. The patient have had two previous transthoracic echocardiographic exams in the last six years and they had only documented moderate aortic regurgitation. Transoesophageal echocardiography displayed a quadricuspid aortic valve. Three cusps were of equal size and one cusp was larger (Figure 1 in diastole and Figure 2 in systole). Planimetric evaluation of the 4 separate cusps performed on an end diastolic still frame resulted in following cusp areas (cm^2^): 1.4, 1.4, 1.5, and 2.2. The three equal cusps had minimal differences in cusp area whereas the larger cusp had a approximately 50% larger cusp area. In this patient the quadricuspid aortic valve malformation was associated with severe aortic regurgitation (Figure 3).

## Discussion

Knowledge of the existence of quadricuspid aortic valve is not new, the first reported case dates back to 1862 [8]. The incidence from autopsy studies matches that detected by echocardiography, around 0.01%, with a slight male predominance [9]. Hurwitz and Roberts introduced a classification nomenclature for quadricuspid aortic valve that included 7 different types named A to G [10]. The type described in this case report, with three equal cusps and one larger, is type E and extremely rare. Tutarel published the most comprehensive review of the literature concerning quadricuspid aortic valve, in 2004 [9]. This review included 186 cases of quadricuspid aortic valve and only 4 was designated type E [9]. Aortic regurgitation is the predominant valvular dysfunction and is seen in up to 75% of documented cases, on the other hand, quadricuspid aortic valve is rarely associated with aortic stenosis [9]. The most prevalent other cardiac malformation associated with quadricuspid aortic valve was anomalies of the coronary arteries, which have been reported in 10% of the cases [9]. Other malformation associated with quadricuspid aortic valve include stenosis of pulmonic valve, nonobstructive cardiomyopathy, subaortic stenosis, and ventricular septal defect [8,9]. Also, cases of bacterial endocarditis affecting a quadricuspid aortic valve have been reported [2,3].

The prevalence of quadricuspid aortic valve is to low to study the diagnostic accuracy of transthoracic versus transoesophageal echocardiography in the detection of this malformation. A recent study by Alegret and co-workers concerning bicuspid aortic valve morphology gives an hint to what might be the case [11]. Alegret et al investigated 59 selected patients and 15 out of 32 bicuspid aortic valves were missed on transthoracic, but detected on transoesophageal echocardiography [11].

## Conclusion

Noteworthy is that two consecutive transthoracic echocardiographic exams had fail to document the quadricuspid aortic valve and this is one more example that liberal use of transoesophageal echocardiography is often warranted if optimal display of valvular morphology is desired. Also, the increasing use of transoesophageal echocardiography will probably lead to an increase in the detection of quadricuspid aortic valve. It is therefore of importance to know this anomaly and its associated defects.

## Competing interests

The author(s) declare that they have no competing interests.

## Authors' contributions

MD did the echocardiography of this case.

MD, MS jointly performed review of the literature and wrote the paper.
